# Does Multi-Fiber-Reinforced Composite-Post Influence the Filling Ability and the Bond Strength in Root Canal?

**DOI:** 10.3390/bioengineering8120195

**Published:** 2021-11-29

**Authors:** Naji Kharouf, Salvatore Sauro, Hamdi Jmal, Ammar Eid, Mohamed Karrout, Nadia Bahlouli, Youssef Haikel, Davide Mancino

**Affiliations:** 1 Department of Endodontics and Conservative Dentistry, Faculty of Dental Medicine, Strasbourg University, 67000 Strasbourg, France; youssef.haikel@unistra.fr (Y.H.); mancino@unistra.fr (D.M.); 2 Department of Biomaterials and Bioengineering, INSERM UMR_S 1121, Strasbourg University, 67000 Strasbourg, France; 3 Dental Biomaterials and Minimally Invasive Dentistry, Department of Dentistry, Cardenal Herrera-CEU University, C/Santiago Ramon y Cajal, s/n., Alfara del Patriarca, 46115 Valencia, Spain; salvatore.sauro@uchceu.es; 4 Department of Therapeutic Dentistry, I. M. Sechenov First Moscow State Medical University, 119146 Moscow, Russia; 5 ICube Laboratory, UMR 7357 CNRS, Mechanics Department, University of Strasbourg, 67000 Strasbourg, France; jmal@unistra.fr (H.J.); mohamed.karrout@etu.unistra.fr (M.K.); bahlouli@unistra.fr (N.B.); 6 Department of Endodontics and Conservative Dentistry, Faculty of Dental Medicine, Damascus University, Damascus 67000, Syria; ammarendo89@gmail.com

**Keywords:** dental materials, scanning electron microscopy, bond strength, post technique

## Abstract

The purpose of the present in vitro study was to investigate the bond strength of root canal dentin and the filling ability of a new multi-fiber-reinforced composite post (mFRC) compared to a conventional single fiber-reinforced-composite post (sFRC). Twenty-eight freshly maxillary first permanent single-rooted premolars were instrumented and divided into groups (*n* = 14). Group 1: single-fiber-reinforced composite (sFRC), group 2: multi-fiber-reinforced composite (mFRC). Bonding procedures were performed using a dual-cure universal adhesive system and resin cement. All specimens were sectioned so that seven discs of 1 mm of thickness were obtained from each root. An optical microscope was used before the push-out test to measure the total area of the voids and to determine the length of the smaller/bigger circumferences. The push-out bond strength (PBS) test was performed using an Instron universal testing machine. Data were then compared by one-way ANOVA on ranks (α = 0.05). The dentin–cement–post interface was observed using scanning electron microscopy (SEM). At the coronal third, a significantly higher bond strength (*p* < 0.05) was obtained in the sFRC group (44.7 ± 13.1 MPa) compared to the mFRC group (37.2 ± 9.2 MPa). No significant difference was detected between the groups at the middle third (sFRC group “33.7 ± 12.5 MPa” and mFRC group “32.6 ± 12.4 MPa”) (*p* > 0.05). Voids were significantly lower in the mFRC compared to those observed in the sFRC group (*p* < 0.05) at the coronal third. Whereas, no significant difference was found at the middle third (*p* > 0.05) between the tested groups. Filling ability was overall improved when employing mFRC, although such technique might have characteristic limitations concerning the bond strength to dentin.

## 1. Introduction

After a successful endodontic treatment, which includes a proper access cavity, shaping, irrigation, and tridimensional filling of the root canal space, clinicians need to select the most appropriate restoration for each endodontically treated tooth (ETT) [1,2,3,4]. Such restorations may often represent a real challenge, especially in teeth that are structurally compromised; several studies have well established that restorative complications represent the main reason for failure, which may likely lead to tooth extraction [5,6]. The analysis of extracted ETT in previous epidemiological studies clearly stated that the vast majority of these were restored without prosthetic cuspal coverage [7,8]. Therefore, coronal coverage significantly improves the clinical success rate of ETT [9]. In such a specific scenario, the use of intra-canal post may be indicated, although the clinical evidence on this specific procedure remains controversial [10]. Indeed, recent clinical studies tend to show a higher success rate for teeth restored with a fiber post [11,12]. However, there is still a lack of clinical data in relation to the material from which the post should be made in order to make clinical recommendations. Superior survival of teeth with rigid or flexible post materials has not been demonstrated [13]. Metallic posts have been widely used for many years [14]. However, these posts have some limitations inherent to root fractures and to aesthetic issues [15]. Their modulus of elasticity is greater than the root dentin, which may be responsible for root fractures [16]. Considering such problems, several researchers have been working to find innovative types of materials for root posts. Generally, fiber posts are made of a bis-GMA or epoxy resin matrix, in which pre-stretched fibers are impregnated; the latter can be made of carbon, glass/silica, and quartz, which the most widely used resin bases are [17]. Over the past decade, the use of single-fiber-reinforced composite (sFRC) to restore tooth integrity has become popular [18]. Their success rate has ranged from 48 to 100% over the years [11,12,15]. Fiber posts present an elasticity modulus similar to that of the root dentin; an aspect that might considerably reduce the risk of root fractures [16,17,18,19,20] along with high aesthetic outcomes [21]. They can be bonded to tooth structures through the use of adhesive systems [17,19]. This combined system transmits the occlusal stress between the root structure and the post, reducing stress concentration and preventing tragic fractures [22,23]. However, bonding of the fiber post to the root is technique-sensitive and also operator-dependent due to the different required clinical steps; this can be considered the weakest link in this type of procedure [24]. Moreover, the placement of sFRC posts often implies extensive removal of the root dentin, which is a major drawback, since tissue preservation is strongly associated with the survival of endodontically treated teeth [25,26]. Several studies [27,28] showed an alternative type of FRC. That is a multi-fiber-reinforced composite (mFRC) based on a bundle of fibers that are bonded directly to the root canal. The application of mFRC into the root canal space does not require any use of post-space preparation [27]. Therefore, the adaptation of mFRC to the root canal anatomy, without additional dentin removal, may be of advantage for tissue preservation.

A typical failure mode in treatment with glass fiber posts (GFP) is often observed as an adhesive failure, as a consequence of the loss of bond strength between the post and the dentin surface [16,19]. Several reasons have been advocated for such a situation. For instance, the inability of the cement to reach and polymerize the deeper areas of the root canal, as well as the shape of the root canal [20,22,29]. In addition, the polymerization shrinkage may generate stress at the bonding interface causing the formations of gaps [23]. Moreover, the formation of gaps generally occurs at the dentin/resin cement interface, since the bond strength in this zone is lower than the bond strength of the resin cement/GFP interface [30]. One of the most relevant ways to observe the resin–dentin interfaces is the use of a scanning electron microscope in order to analyze the voids (microorganism’s pathway) and to observe the resin infiltrations (tags) into dentinal tubules [31].

The purpose of the present in vitro study was to investigate the bond strength to root canal dentin and the filling ability of a new multi-fiber-reinforced composite post compared to a conventional single fiber-reinforced composite post. The first null hypothesis was that there is no difference between the two post systems among filling ability and the second one was that the mFRC would have no significant impact on the bond strength in the root canal.

## 2. Materials and Methods

### 2.1. Sample Preparation

Forty freshly maxillary first permanent premolars were obtained under patient-informed consent. The teeth were extracted for orthodontic reasons (18–24 years old), single canal, with a fully formed root canal, and with a total length between 21 and 23 mm. The ethics Committee of Medical, Odontology School, Strasbourg University Hospital approved the protocol (Protocol No. 2019-05). The samples were immersed in saline solution no longer than 30 days after debridement of the root surface.

Cone beam computed tomography (CBCT) was used to preselect the teeth respecting the following criteria:

1. Single canal;
2. A long/short canal diameter ratio at 5 mm from the apex >2 [32];
3. The length of root canal (orifice to apical foramen) = 14 ± 1 mm;
4. Primary root curvature ≤20 in bucco-lingual and mesio-distal view [33];
5. Main curvature radius ≥4 mm.

After selection, 28 teeth were finally included in the experimental design (12 teeth were excluded).

The teeth were decoronated using a diamond saw (Well Walter Ebner, Manheim, Germany) 1 mm above the cemento-enamel junction. In addition, a constant reference point was obtained at 15 ± 1 mm. The same operator performed all the endodontic steps. After scouting and glide path, a # 10 K file was used to determine the working length (WL) under a microscope (Zumax Medical Co., Ltd., Suzhou, Jiangsu, China) by subtracting 1 mm from the length at the apical foramen. The root canals were instrumented with rotary nickel-titanium instruments Plex v (Orodeka, Shandong, China), 15/03 (1.5 N.CM, 300 rpm), 20/05, 25/04, 30/04 (2.5 N.Cm, 500 rpm) accompanied with 3 mL of 5.25% sodium hypochlorite using 31-gauge Navitip needles and the apical patency rechecked. After that, the canals were rinsed with distilled water and dried with paper points. The roots were obturated with CWC using a 30/04 adjusted gutta-percha cone with an endodontic sealer (Sealapex, Kerr Dental France, Ivry-sur-Seine, France).

### 2.2. Post Placement

The samples were randomly divided into two equal groups (n = 14 for each post type):

Group 1: single fiber-reinforced composite (sFRC) (Biolight, Tullins, France)

Group 2: multi-fiber-reinforced composite (mFRC) (Biolight, Tullins, France)

Concerning the first group (sFRC), 7 mm of the coronal side of the canal was prepared using #1 reamer (Bioligth, Tullins, France) as measured from the cementum–enamel junction on the buccal aspect of the tooth. Conversely, for group 2, no post space preparation was required and the 7 mm of obturation were removed using an ultrasonic #25K-file (SATELEC Equipement dentaire, Aquitaine, France). For both techniques, the canal was etched using 37% phosphoric acid (Itena Clinical, Paris, France) for 30 s. Then, the canal was rinsed with distilled water and dried using a gentle air stream, followed by the use of paper points.

Bonding procedures were performed using a dual-cure universal adhesive system [34] and resin cement (CLEARFIL core build-up Kit, Kuraray Europe GmbH, Hattersheim, Germany). The resin cement was delivered into the root canal space using a lentulo tip, followed by the placement of a single post. For the mFRC, posts were inserted manually into the root canal. The excess resin was removed and the light-curing procedure was performed through the post for 20 s using a Luxite Lampe LED (Itena Clinical, Paris, France).

The teeth were then embedded in epoxy resin using a cylindrical silicone mold (20 mm in height; 10 mm in diameter). Eight sections were made perpendicularly to the tooth axes using a precision cutting machine (MICRACUT 152, Metkom, Bursa, Turkey) under continuous water cooling. Seven discs of 1 mm of thickness were obtained from each tooth.

### 2.3. Filling Ability

**Figure 1:**
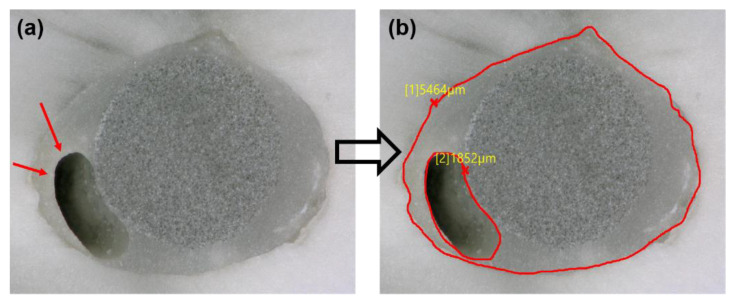
(**a**) Representative photos obtained with a digital microscope of sectioned root surfaces with the presence of void (red arrows); (**b**) methodology of void measurements.

Both surfaces of each disc were observed using an optical microscope (KEYENCE, Osaka, Japon) at 50× magnification. The VHX-5000 communication software (KEYENCE, Osaka, Japon) was used to determine the length of the smaller/bigger circumferences. The same software was used to measure the void percentages by dividing the void area on the filled root canal area (Figure 1).

### 2.4. Push-Out Test

**Figure 2:**
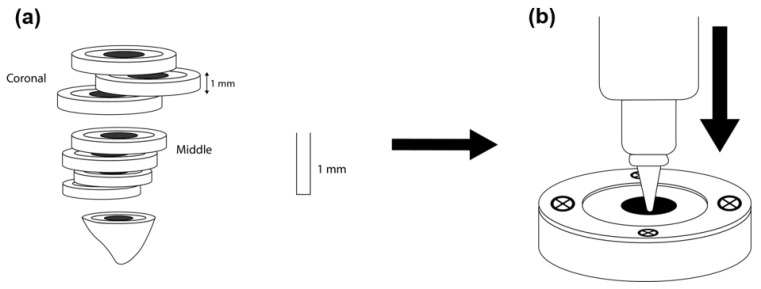
(**a**) Stainless steel pluggers of diameters used according to the root canal third to perform the push-out test. (**b**), push-out test using an Instron universal tension/compression machine.

The push-out test, based on a methodology previously published [35], was used to determine the bond strength. A 1 mm stainless steel plugger [36] was used according to the root canal third (middle or coronal) to perform the push-out test. The push-out was applied using an Instron universal testing machine (Instron 3345, High Wycombe, UK). The machine was equipped with a 1 kN load cell (Instron 2519-1 kN) and a controller for displacement. The plugger was initially positioned near the interface without touching the specimen. The force value was then set to zero. The tap displacement was set at a rate of 1 mm/min until the failure occurred in the apico-coronal direction of each disc (Figure 2). Force values were recorded during tests using the Bluehill^®^ universal software (Version 4.03). The bond strength was determined in megapascals (MPa), which was calculated based on the following formula:

N = F/S

where N = bond strength (MPa); F = maximum load (N), and S = adhesion area of root canal (mm^2^).

The adhesion area of the root canal was measured using the following formula:

S = ½ a h^2^ + b h

a = (bigger circumference–smaller circumference)/h

b = smaller circumference

h = the thickness of the section (1 mm)

### 2.5. Failure Mode Analysis

After the push-out test, an optical microscope (Keyence, Osaka, Japan) was used to investigate the failure mode in each specimen at 200× magnification. The VHX-5000 software was used to calculate the percentage of each area to define the type of failure. The failures were categorized [35] into (i) adhesive failure between the dentin and cement and/or between the cement and fiber post; (ii) cohesive failure within the fiber post, dentin, or cement material; (iii) mixed failure.

### 2.6. Scanning Electron Microscopy Observation (SEM)

Finally, two samples from each third (middle and coronal) of each group were prepared for SEM analysis. Subsequently, the surface of the sample was polished using SiC abrasive discs (1200, 2400, and 4000). Thirty-seven percent phosphoric acid was applied on the polished surfaces for 5 s, followed by 2.5% NaOCl (3 min) in order to treat the smear layer and to observe the sealer tags in dentinal tubules (infiltrations) [2]. After the dehydration step using a graded series of ethanol solutions (50%, 75%, 95%, and 100%), the samples were sputter-coated with a gold–palladium alloy (20/80 weight %) using a sputtering device (Technics, CA, USA). Finally, the samples were observed using a Quanta 250 FEG scanning electron microscope “SEM” (FEI Company, Eindhoven, The Netherlands, 10 kV) with a working distance of 10 mm to observe the dentin–cement–post interfaces.

### 2.7. Statistical Analysis

The normality of data within both groups was verified using the Shapiro–Wilk test. One-way analysis of variance (ANOVA) including multiple comparison procedures (Holm–Sidak method and Tukey Test) was applied to determine whether significant differences existed between the different fiber posts for the bond strength values and void percentages. Sigma Plot (11.2, Systat Software, Inc., San Jose, CA, USA) was used for data analyses with a significance level at α = 0.05.

## 3. Results

The normality of data within both groups was passed for all tests. At the coronal third, the sFRC group showed a statistically greater bond strength (44.7 ± 13.1 MPa) compared to the mFRC group (37.2 ± 9.2 MPa) (*p* < 0.05), while no significant difference was found between the sFRC group (33.7 ± 12.5 MPa) and the mFRC group (32.6 ± 12.4 MPa) (*p* > 0.05) at the middle third.

**Figure 3:**
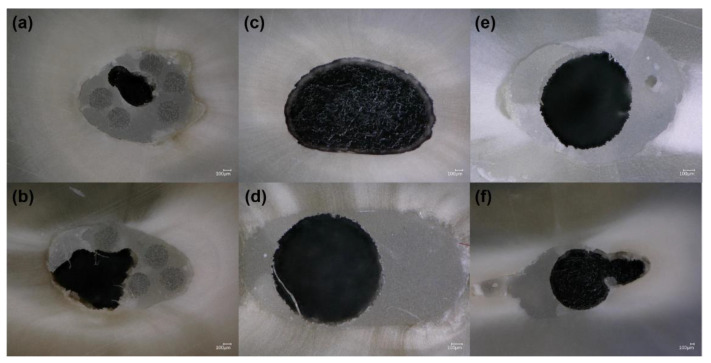
Representative images of optical microscopy (Keyence, Osaka, Japan). Failure analysis of (**a**,**b**) mixed failure in mFRC group; (**c**–**e**) adhesive failure, and (**f**) mixed failure in sFRC group.

Optical observations after the push-out test revealed that, in the case of the mFRC group, most of the specimen fractures occurred between cement, dentin, and micro-posts (mixed failure) (Figure 3a,b). The (sFRC) group showed mostly an adhesive failure between the cement and root dentin (Figure 3c); as well as between cement and the post (Figure 3d,e) and some mixed failures (Figure 3f).

**Figure 4:**
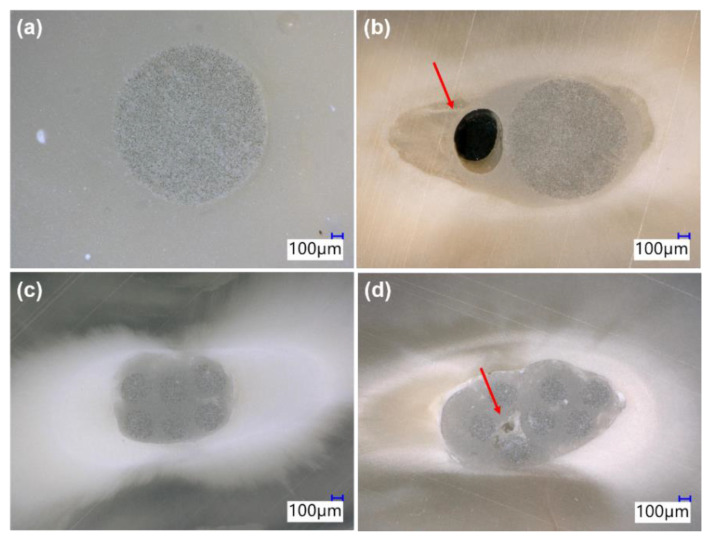
Representative images obtained with a digital microscope. (**a**,**b**) sFRC, (**c**,**d**) mFRC showing internal voids (arrow).

A significantly lower void percentage was observed with mFRC compared to those for the sFRC group (*p* < 0.05) at the coronal third (Figure 4, Table 1), whilst no significant difference was found at the middle third (*p* > 0.05).

**Figure 5:**
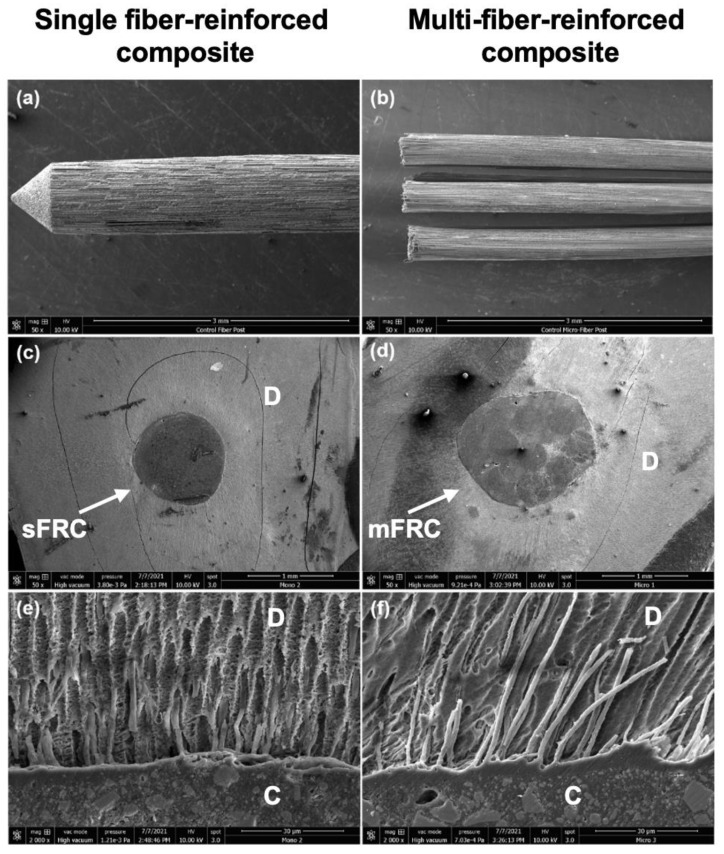
Representative photos of scanning electron microscopy of: (**a**) sFRC; (**b**) mFRC; (**c**) sFRC–cement–dentin interfaces; (**d**) mFRC–cement–dentin interfaces; (**e**,**f**) infiltrations into dentinal tubules.

Figure 5b,c shows the fiber post/dentin/cement interfaces. Regarding cement infiltrations into the dentinal tubules, tags were observed for both groups; thus, the dentinal tubules were occluded by the cement materials in both groups (Figure 5e,f).

## 4. Discussion

This study demonstrated that both types of fiber posts may produce the same level of voids in the middle parts of the root canal, but at the coronal third of the root canal, the multi-fiber-reinforced composite can have a much better adaptation with less risk for void formation. Therefore, the first null hypothesis must be rejected. In the present study, the filling ability was associated with the post geometry, microfiber distribution, and the dimension of the filled canal. At the coronal third (larger canal third) the geometry and the distribution of mFRC could play an important role in the filling quality of the root canal. However, such voids were mainly observed for single fiber-reinforced composite inside the filling materials “closed pores” (Figure 4b) rather than at the bonding interface. It has been advocated that voids may serve as a pathway for bacteria penetration from the coronal area of the restoration to the apex of the root, causing the reinfection of the root canal system and the failure of the treatment [2]. Moreover, Uzun et al. [37] reported that the quality of filling ability in coronal third may play an important role among mechanical risks in this area such as root and restoration fractures. The same authors also observed a larger and more significant presence of voids at the coronal third compared to the middle third; the results of the current study are in agreement with Uzun et al. [37].

A push-out test was used in this study in accordance with other relevant studies published in the dental literature [38]. According to the filling ability results, it was expected that the bond strength of sFRC could be lower than the mFRC group. In contrast, in the present study, bond strength results at the coronal third showed a higher significant difference for the sFRC group than the mFRC group (*p* < 0.05). Therefore, the second null hypothesis must be rejected. However, in the middle third, no significant difference was found among the push-out test between the tested fiber post groups. The indenter diameter used for the push-out test was approximately 1 mm, which allowed us to apply a local force at the coronal and middle third, accurately. For the sFRC group, the local force was applied directly on the post which is “macro-post” with a coronal diameter at a minimum of 1.25 mm. Thus, the resistance of the fiber post is transmitted to the indenter and in several samples, only the interface between post-cement is loaded. Optical observations of the group sFRC specimens at coronal third, after push-out the test revealed that most of the specimens failed between fiber post and cement (Figure 3d,e), whilst the mFRC group mostly showed at coronal third a mixture failure mode (Figure 3a,b). The force at coronal third for the mFRC group is applied on a surface composed of micro-posts and cement. In this case (mFRC at coronal third), during the push-out test, the applied force produces micro-fissures which are limited probably in the cement-micro-post area and do not reach the cement-dentin interface. For these reasons discussed above, the strength stress of the sFRC group was higher than the mFRC group at the coronal third, which indicates that there may be a good interaction of the resin cement with the post fiber [39]. In contrast, at the middle third, the anatomy shape of the root canal is conduct to have a low distribution of micro-post at the middle third compared to the coronal third. Therefore, the cement spaces between the microfiber (weak area) are smaller than at coronal third. This fact conducts the microfiber to construct a cluster with similar behavior to a single post. Therefore, at the middle third, the push-out force is applied on post for both mFRC and sFRC groups. In addition, the statistical results show no difference between mFRC and sFRC groups in the middle third. Moreover, the optical observation shows that the principal failure mode for both groups at the middle third is adhesive failure.

The application of mFRC into the root canal space does not require any use of post-space preparation [27]. Therefore, the adhesive area between mFRC and root canal dentin could be smaller than the adhesive area between sFRC and root canal. The interfaces between both systems with root canal dentin were similar following the SEM observations and both systems could create resin infiltrations into dentinal tubules. The penetration of the sealers into the dentinal tubule may represent an important factor to entomb the bacteria [1,2] and to support an interaction between the sealer materials and the dentinal fluid.

The most common factor of the fail of dental restorations is fatigue. The simulation of the fatigue of dental restorations in clinical service could be made in vitro by thermomechanical cycling [40,41]. However, the stability of bond strength of a resin adhesive bonded to tooth tissues in the oral cavity is an important aspect to promote optimal dental restoration. In addition, a severe degradation over time of the resin–dentin interface and the increase in nanoleakage lead to limited bond durability [42,43].

Santos et al. [28] evaluated the fracture resistance of conventional and bundled glass-fiber-reinforced composite posts in bovine incisors. Their results, after loading to fracture, showed that the combination between the two post systems could improve the stress distribution and the resistance of immature teeth. However, in their study, post space preparation was performed for the two groups and the specimens used were bovine incisors which have a pulp canal area larger than human incisors.

One limitation of this study was that the push-out test was performed immediately without aging and fatigue. Therefore, further analysis should be performed in order to evaluate the impact of aging and fatigue on the bond strength of fiber post to dentin. The optical microscopy performed in this study has limitations, and only a small number of sections could be examined using such destructive techniques. Therefore, further analysis is required using µCt microscopy in order to investigate the whole canal in 3D observations. Moreover, concerning the push-out test, the apical third of each canal was not accessible for the plugger tips. Furthermore, within the limitations of the push-out test as mentioned above, microtensile bond strength test seems to be more recommended to analyze the interaction between fiber post/cement/dentin in the root canal [44]. Further studies are required to analyze the different chemical pre-treatments of sFRC and/or mFRC on their bond strength to the cement or root canal dentin.

## 5. Conclusions

The mFRC demonstrated superior results in filling ability compared to sFRC. However, the bonding ability to dentin of mFRC may be inferior at the coronal third compared to sFRC.

## Figures and Tables

**Table 1:** Void percentages at middle and coronal third.

	sFRC	mFRC	*p* < 0.05
Coronal (%)	14.6 ± 9.4	5.8 ± 2.6	Yes (*p* = 0.011)
Middle (%)	4.2 ± 9.03	1.3 ± 3.2	No (*p* = 0.356)

## Data Availability

Not applicable.
